# Effect of Nondominant Left-Handed Phacoemulsification Surgery on Corneal Endothelium

**DOI:** 10.7759/cureus.36744

**Published:** 2023-03-27

**Authors:** Mehmet İçöz, Şule Gökçek Gürtürk İçöz, Mücella Arıkan Yorgun

**Affiliations:** 1 Department of Ophthalmology, Yozgat City Hospital, Yozgat, TUR; 2 Department of Ophthalmology, Yildirim Beyazit University Faculty of Medicine, Ankara, TUR

**Keywords:** cataract, endothelial cell, cornea, nondominant hand, phacoemulsification

## Abstract

Purpose: To evaluate the effect of phacoemulsification surgery performed with the nondominant left hand on the corneal endothelium.

Methods: The study included 160 patients who were followed up for at least six months after uneventful cataract surgery. Seventy-seven patients who underwent nondominant left-handed phacoemulsification were evaluated as Group 1 and 83 patients who underwent dominant right-handed phacoemulsification were evaluated as Group 2. In all the patients, preoperative axial length (AL), aqueous depth (AD), anterior chamber depth (ACD), and lens thickness (LT) were evaluated. All phacoemulsification procedures were undertaken with the same device by two surgeons using the same technique, and the intraoperative cumulative dissipated energy (CDE) value, effective phacoemulsification time, and total operative time were recorded. Corneal endothelial cell density (ECD), coefficient of variation (CV) percentage, hexagonal cell percentage (HCP), and central corneal thickness (CCT) measurements were evaluated by specular microscopy preoperatively and at the postoperative first and sixth months.

Results: The two groups had a similar age and gender distribution and did not significantly differ in terms of the preoperative AL, AD, ACD, LT, intraoperative CDE, effective phacoemulsification time, and total operative time with similar age and gender distribution (p > 0.05 for all). The preoperative and postoperative first- and sixth-month specular microscopy measurements of ECD, CV, HCP, and CCT ​​were also similar in the groups (p > 0.05 for all).

Conclusions: In this study, it was observed that nondominant left-handed phacoemulsification resulted in similar changes in endothelial cell count and morphology to those obtained from dominant-handed phacoemulsification.

## Introduction

Cataract surgery is one of the most frequently performed ophthalmological operations [1]. Phacoemulsification is a procedure that requires hand-foot-eye coordination and a certain learning curve, and surgeons mostly use their dominant hand during the critical phases of surgery [2]. The mean number of endothelial cells in an adult human is between 2,000 and 2,500 cells/mm^2^. It is known that with increasing age, the number of endothelial cells decreases by an average of 0.3-0.5% per year [3]. In addition to aging, endothelial cell loss occurs after intraocular operations. Depending on this loss, there may also be changes in endothelial cell morphology and density [4,5].

In the literature, various studies have reported a decrease in endothelial cell density (ECD), an increase in the coefficient of variation (CV) percentage, and a decrease in the number of hexagonal cells after cataract surgery [6,7]. It is known that during phacoemulsification, excessive ultrasound energy, contact of lens pieces with the corneal endothelium, air bubbles, and increased localized temperature can cause endothelial cell damage [8-11].

This study aimed to evaluate the effect of nondominant left-handed phacoemulsification surgery on corneal endothelial cell loss and morphological changes.

## Materials and methods

This cross-sectional observational study was performed at a tertiary ophthalmology clinic from January to December 2021, with approval from the Ankara City Hospital Ethics Committee (approval number: E1-23-3238). The study adhered to the tenets of the Declaration of Helsinki and informed consent was obtained from all the participants.

The study included 77 patients who underwent uneventful cataract surgery with nondominant left-handed phacoemulsification and 83 patients who underwent dominant right-handed phacoemulsification. The exclusion criteria were the presence of cataracts other than senile cataracts, glaucoma, uveitis, trauma, history of any eye surgery or ocular disease, diabetes, and a preoperative endothelial cell count below 2,000 cells/mm^2^. In addition, the cases of radial tear in the anterior capsule, zonular weakness, posterior capsule rupture, and dropped nucleus, which could develop intraoperatively, and patients with postoperative intense or persistent corneal edema, anterior chamber reaction, wound leakage, posterior capsule opacity, and macular edema were not included in the study.

All the patients underwent an ophthalmological examination. Using a slit lamp, lens nucleus opacification was evaluated, and cataracts were classified as soft, medium-hard, very hard, and mature. Axial length (AL), aqueous depth (AD), anterior chamber depth (ACD), and lens thickness (LT) were recorded using the LenStar biometry 900 (Haag-Streit, Köniz, Switzerland) designed for preoperative intraocular lens measurement. Cumulative dissipated energy (CDE) applied during phacoemulsification surgery, effective phacoemulsification time, and total operative time were recorded. The corneal endothelial cell count, CV, hexagonal cell percentage (HCP), and central corneal thickness (CCT) values were determined preoperatively and at the postoperative first and sixth months using a specular microscopy device (Cem-530, Nidek Co., Ltd, Gamagori, Japan).

All the cataract operations were performed by two surgeons (M.I. and S.G.G.I.), with the patients placed under topical anesthesia. For phacoemulsification, a 2.2-mm clear corneal incision was made with the stop-and-chop technique. In both eyes, surgery was performed from the superior temporal quadrant. In all the patients, low-viscosity viscoelastic material was used in the capsulorhexis stage and high-viscosity viscoelastic material in other stages. For phacoemulsification, the Centurion system (Alcon, Fort Worth, TX) device was used, and the Alcon SA60AT single-piece, foldable, monofocal intraocular lens (Alcon, Fort Worth, TX) was placed in the capsular bag.

The Statistical Package for the Social Sciences (SPSS) version 22 for Windows (IBM Corp., Armonk, NY) was used for statistical analyses. Quantitative values ​​were expressed as mean ± standard deviation. The conformance of the data to the normal distribution was evaluated visually by histograms and statistically by the Kolmogorov-Smirnov test. The lens nucleus score was compared between the two groups using the Pearson chi-square test. Continuous data were compared between two groups with the independent-sample t-test since data distribution met the assumption of normality. A p-value of <0.05 was considered statistically significant.

## Results

The study included a total of 160 patients who underwent uneventful cataract surgery and were followed up for six months. Of these patients, 77 who underwent nondominant left-handed phacoemulsification were evaluated as Group 1, and 83 who underwent dominant right-handed phacoemulsification were evaluated as Group 2. The age and gender distributions of the groups were similar (p = 0.45 and p = 0.63, respectively). There was also no statistically significant difference between the two groups in terms of the preoperatively measured anterior segment parameters of AL, AD, ACD, and LT and the intraoperative CDE, total operative time, and effective phacoemulsification time (p > 0.05 for all). The p-values ​​are shown in Table 1.

**Table 1:** Distribution of the demographic, preoperative, and intraoperative parameters between the groups Group 1: nondominant left-handed phacoemulsification; Group 2: dominant right-handed phacoemulsification; AL: axial length; AD: aqueous depth; ACD: anterior chamber depth; LT: lens thickness; CDE: cumulative dissipated energy. * Mann-Whitney U test; mean ± standard deviation.

Parameter	Group 1 (n = 77)	Group 2 (n = 83)	p-value*
Age (years)	65.3 ± 8.3	64.3 ± 9.2	0.45
Female (n - %)	40 (51.9%)	39 (46.9%)	0.63
AL (mm)	23.2 ± 0.82	23.3 ± 0.89	0.65
AD (mm)	2.7 ± 0.36	2.8 ± 0.35	0.08
ACD (mm)	3.2 ± 0.36	3.3 ± 0.35	0.09
LT (mm)	4.2 ± 0.40	4.3 ± 0.41	0.15
CDE (%-second)	6.2 ± 2.7	6.6 ± 3.5	0.43
Total operative time (min)	17.5 ± 3.5	18.2 ± 2.9	0.34
Effective phacoemulsification time (min)	6.1 ± 1.5	5.8 ± 2.0	0.28

According to the lens nucleus opacification evaluation, the cataracts were divided into four stages: soft, medium-hard, very hard, and mature. The groups were evaluated according to these stages with the Pearson chi-square test. There was no statistically significant difference between the groups in terms of cataract hardness (Pearson x2 = 0.51, p = 0.91) (Table 2).

**Table 2:** Distribution of lens nucleus opacification by groups Group 1: nondominant left-handed phacoemulsification; Group 2: dominant right-handed phacoemulsification.

Lens nucleus opacification	Group 1 (n = 77)	Group 2 (n = 83)	Pearson chi-square	p-value
Soft	9	9	0.51	0.91
Medium hard	32	39		
Very hard	26	26		
Mature	10	9		

Table 3 presents the comparison of Groups 1 and 2 in terms of the preoperative and postoperative first- and sixth-month corneal specular microscopy measurements, namely, ECD, CV percentage, HCP, and CCT.

**Table 3:** Comparison of preoperative and postoperative first- and sixth-month specular microscopy measurements between the two groups Group 1: nondominant left-handed phacoemulsification; Group 2: dominant right-handed phacoemulsification; ECD: endothelial cell density; CV: coefficient of variation; HCP: hexagonal cell percentage; CCT: central corneal thickness. * Mann-Whitney U test; mean ± standard deviation.

Parameter	Group 1 (n = 77)	Group 2 (n = 83)	p-value*
Preoperative ECD (cell/mm^2^)	2630 ± 330	2595 ± 307	0.48
Postoperative first-month ECD (cell/mm^2^)	2316 ± 36	2318 ± 35	0.97
Postoperative sixth-month ECD (cell/mm^2^)	2221 ± 34	2229 ± 32	0.86
Preoperative CV (%)	33.3 ± 5.4	33.2 ± 5.0	0.91
Postoperative first-month CV (%)	35.2 ± 0.64	35.0 ± 0.62	0.77
Postoperative sixth-month CV (%)	36.4 ± 0.46	36.1 ± 0.62	0.40
Preoperative HCP (%)	66.1 ± 5.3	66.1 ± 4.2	0.91
Postoperative first-month HCP (%)	65.1 ± 0.62	65.0 ± 0.65	0.91
Postoperative sixth-month HCP (%)	65.5 ± 0.45	64.9 ± 0.48	0.34
Preoperative CCT (µm)	543 ± 29	548 ± 34	0.39
Postoperative first-month CCT (µm)	554 ± 35	547 ± 36	0.22
Postoperative sixth-month CCT (µm)	550 ± 35	543 ± 33	0.16

## Discussion

Cataract surgery is one of the most frequent ocular operations performed by ophthalmologists and requires a certain learning curve. Most surgeons use their dominant hand during the critical phases of surgery. To minimize the patient’s postoperative astigmatism, a superotemporal or perpendicular incision is recommended [1]. Various studies have investigated factors affecting endothelial cell loss after cataract surgery [2,12,13]. In the current study, we aimed to evaluate the effect of phacoemulsification performed with the nondominant left hand on endothelial cell count and morphology.

Sharma et al. compared cataract operations performed with the nondominant and dominant hands, in which the surgeon was seated at the head end or the temporal side. Phacoemulsification power, effective phacoemulsification time, surgery-induced astigmatism, endothelial cell loss, and visual acuity were found to be similar between the four groups [2]. Kageyama et al. evaluated a total of 410 patients who underwent cataract surgery performed by four young ophthalmologists using the dominant hand (n = 203) or nondominant hand (n = 207). The authors reported the complication rate to be 19.7% in dominant-hand phacoemulsification surgery and 14.5% in nondominant-hand phacoemulsification surgery, indicating no statistically significant difference between the two groups. Kageyama et al.'s study was conducted on ophthalmology residents. The high rate of these complications may be due to young ophthalmologists. In the same study, the patients were divided into four subgroups to evaluate the effect of phacoemulsification surgery performed with the dominant and nondominant hand on changes in corneal endothelial cell count, and the procedure provided similar results in all the groups [12].

In this study, in addition to ECD, we also evaluated changes in CV, HCP, and CCT and found that these parameters similarly changed in the postoperative period in both groups. Surgeons using their dominant hand change their position to perform surgery at the superotemporal or temporal main incision. This also changes the location of the phacoemulsification device, surgeon’s chair, and operating microscope. Perone et al. suggested that CCT after cataract surgery might be one of the important indicators of endothelial cell damage [13]. In our study, corneal thickness increased in the postoperative period and was accompanied by endothelial cell loss. However, phacoemulsification surgery performed with the dominant and nondominant hand resulted in a similar increase in corneal thickness. Lucena et al. compared phacoemulsification operations performed using a balanced salt solution (BSS) Plus and Ringer’s lactate irrigation solution. They reported a decrease in endothelial cell count, an increase in CV, and no significant change in CCT values in either group at the second-month follow-up. The authors noted that these evaluated parameters did not significantly differ between the two groups. In the current study, while the CCT values ​​increased in both groups at the sixth-month follow-up compared to the preoperative evaluation, there was no significant difference between the groups. Changes in the CV value and endothelial cell count were consistent with those reported by Lucena et al. [14].

It is known that after cataract surgery, there is an endothelial cell loss of 4% and 25% [15]. This loss depends on many different preoperative and intraoperative factors. Ganekal et al. evaluated the endothelial cell count and morphological characteristics of patients who underwent phacoemulsification. They found the endothelial cell count of the patients as 2323 cells/mm^2^ preoperatively and 2247 cells/mm^2^ at the postoperative sixth week. In addition, the preoperative CV increased from 38% preoperatively to 42% in the postoperative sixth week. Lastly, the CCT was determined as 578 microns preoperatively and 574 microns at the postoperative sixth week [16]. Bozkurt Oflaz et al. evaluated the surgical training of 66 participants with a cataract surgery simulator and compared the results of capsulorhexis performed with the nondominant hand according to surgeon experience. The authors reported that the success of nondominant hand capsulorhexis increased as surgical experience increased [17].

Considering that approximately 10% of the world population is left-handed, right-hand dominance would be similarly observed among ophthalmologists [18]. Anderson et al. reported that left-handed surgeons had more difficulty and were less comfortable when performing surgery [19]. Therefore, Mukherjee et al. stated that manual small incision cataract surgery, which can be applied as an alternative to phacoemulsification surgery, was a more safe and easier method that could be employed by left-handed surgeons [18]. It is difficult to perform surgery with the left hand, and it can be difficult to transfer surgical skills to young ophthalmologists in training clinics. As left-handed phacoemulsification surgery practice increases, the surgeon’s self-confidence will also increase, and the training process of left-handed young ophthalmologists will be less challenging.

Various studies have evaluated endothelial cell loss, pleomorphism, polymegathism, and CCT after phacoemulsification surgery [15,16]. It is known that the dominant hand is mostly preferred during phacoemulsification surgery. In the current study, it was determined that the effect of nondominant hand phacoemulsification surgery on endothelial cells and their properties was similar to that of dominant-hand phacoemulsification surgery. After a certain learning curve, phacoemulsification surgery can be performed with the dominant or nondominant hand at similar efficacy. During phacoemulsification surgery, by placing the phaco handpiece in the nondominant hand, the chopper can be used more actively in the dominant hand. This facilitates the effective rupture and fragmentation of the nucleus and allows for a more controlled and reliable phacoemulsification procedure. We consider that the use of both hands ensures the more effective application of phaco chop techniques, eliminates the need to change the sitting position during surgery, and increases the self-confidence of the surgeon.

Since pterygium tissue is mostly seen in the nasal region [20], when performing left-eye cataract surgery, corneal incisions made close to the pterygium tissue can be difficult to close with stromal hydration at the end of surgery. In these cases, if the main incision for the left eye is made in the superotemporal quadrant and the lateral entry points are opened in the superonasal quadrant, complications that may develop due to the entry sites can be seen less frequently.

Among the main limitations of the study are the single-center design, operations being performed by two surgeons, and the absence of longer-term results. In addition, although the evaluation of lens nucleus opacification was performed subjectively, it was observed that the two groups were similar to each other. Furthermore, we only evaluated corneal endothelial count and morphology, but the inclusion of intraoperative and postoperative complications and postoperative refraction results could offer a better insight into which hand can achieve more reliable surgery. There is a need for multicenter studies with a larger number of patients and multiple surgeons to contribute to the literature.

## Conclusions

In this study, it was observed that nondominant left-handed phacoemulsification surgery resulted in similar changes in endothelial cell count and morphology to those obtained from dominant-handed phacoemulsification surgery. Therefore, phacoemulsification surgery can be effectively and safely performed with either hand.

## References

[1] Solborg Bjerrum S, Mikkelsen KL, la Cour M (2015). Epidemiology of 411 140 cataract operations performed in public hospitals and private hospitals/clinics in Denmark between 2004 and 2012. Acta Ophthalmol.

[2] Sharma V, Sinha R, Sharma N, Dada T, Tandon R, Titiyal JS, Vajpayee RB (2007). Phacoemulsification with nondominant hand. Eye (Lond).

[3] Yee RW, Matsuda M, Schultz RO, Edelhauser HF (1985). Changes in the normal corneal endothelial cellular pattern as a function of age. Curr Eye Res.

[4] Batlan SJ, Dodick JM (1996). Corneal complications of cataract surgery. Curr Opin Ophthalmol.

[5] Demir AG, Olgun A, Guven D, Demir M, Sendul SY, Akarsu Acar OP, Kacar H (2019). The effect of combined phacotrabeculectomy, trabeculectomy and phacoemulsification on the corneal endothelium in the early stage: a preliminary study. Int Ophthalmol.

[6] Schultz RO, Glasser DB, Matsuda M, Yee RW, Edelhauser HF (1986). Response of the corneal endothelium to cataract surgery. Arch Ophthalmol.

[7] Ventura AC, Wälti R, Böhnke M (2001). Corneal thickness and endothelial density before and after cataract surgery. Br J Ophthalmol.

[8] Pirazzoli G, D'Eliseo D, Ziosi M, Acciarri R (1996). Effects of phacoemulsification time on the corneal endothelium using phacofracture and phaco chop techniques. J Cataract Refract Surg.

[9] Miyata K, Nagamoto T, Maruoka S, Tanabe T, Nakahara M, Amano S (2002). Efficacy and safety of the soft-shell technique in cases with a hard lens nucleus. J Cataract Refract Surg.

[10] Kim EK, Cristol SM, Kang SJ, Edelhauser HF, Yeon DS, Lee JB (2002). Endothelial protection: avoiding air bubble formation at the phacoemulsification tip. J Cataract Refract Surg.

[11] Sippel KC, Pineda R Jr (2002). Phacoemulsification and thermal wound injury. Semin Ophthalmol.

[12] Kageyama T, Yaguchi S, Metori Y, Chida M, Koizumi K, Onishi T, Ayaki M (2002). Visual results and complications of temporal incision phacoemulsification performed with the non-dominant left hand by junior ophthalmologists. Br J Ophthalmol.

[13] Perone JM, Boiche M, Lhuillier L (2018). Correlation between postoperative central corneal thickness and endothelial damage after cataract surgery by phacoemulsification. Cornea.

[14] Lucena DR, Ribeiro MS, Messias A, Bicas HE, Scott IU, Jorge R (2011). Comparison of corneal changes after phacoemulsification using BSS Plus versus lactated Ringer's irrigating solution: a prospective randomised trial. Br J Ophthalmol.

[15] Mencucci R, Ponchietti C, Virgili G, Giansanti F, Menchini U (2006). Corneal endothelial damage after cataract surgery: microincision versus standard technique. J Cataract Refract Surg.

[16] Ganekal S, Nagarajappa A (2014). Comparison of morphological and functional endothelial cell changes after cataract surgery: phacoemulsification versus manual small-incision cataract surgery. Middle East Afr J Ophthalmol.

[17] Bozkurt Oflaz A, Ekinci Köktekir B, Okudan S (2018). Does cataract surgery simulation correlate with real-life experience?. Turk J Ophthalmol.

[18] Mukherjee A, Kalamkar C, Rao R, Patel B (2022). Technique modifications in manual small-incision cataract surgery for left-handed cataract surgeons. Indian J Ophthalmol.

[19] Anderson M, Carballo E, Hughes D, Behrer C, Reddy RM (2017). Challenges training left-handed surgeons. Am J Surg.

[20] Lotfy A, Gad AA, Abdelrahman A, Samir A, Abdulhalim BH (2018). Conjunctival autograft combined with either preoperative mitomycin c injection or intraoperative local mitomycin c over the medial rectus muscle tendon in primary pterygium surgery. Eye Contact Lens.

